# Music playschool enhances children’s linguistic skills

**DOI:** 10.1038/s41598-018-27126-5

**Published:** 2018-06-08

**Authors:** Tanja Linnavalli, Vesa Putkinen, Jari Lipsanen, Minna Huotilainen, Mari Tervaniemi

**Affiliations:** 1 Cognitive Brain Research Unit, Faculty of Medicine, University of Helsinki, P.O.Box 9 FIN-00014 University of Helsinki, Helsinki, Finland; 2 Turku PET Centre, University of Turku, Turku, Finland; 3 Psychology and Logopedics, Faculty of Medicine, University of Helsinki, Helsinki, Finland; 4 Cicero Learning, Faculty of Educational Sciences, University of Helsinki, Helsinki, Finland

## Abstract

Several studies have suggested that intensive musical training enhances children’s linguistic skills. Such training, however, is not available to all children. We studied in a community setting whether a low-cost, weekly music playschool provided to 5–6-year-old children in kindergartens could already affect their linguistic abilities. Children (N = 66) were tested four times over two school-years with *Phoneme processing* and *Vocabulary* subtests, along with tests for *Perceptual reasoning skills* and *Inhibitory control*. We compared the development of music playschool children to their peers either attending to similarly organized dance lessons or not attending to either activity. Music playschool significantly improved the development of children’s phoneme processing and vocabulary skills. No such improvements on children’s scores for non-verbal reasoning and inhibition were obtained. Our data suggest that even playful group music activities – if attended to for several years – have a positive effect on pre-schoolers’ linguistic skills. Therefore we promote the concept of implementing regular music playschool lessons given by professional teachers in early childhood education.

## Introduction

Considerable amount of research suggests that practicing musical skills can contribute to linguistic abilities. Previous studies have found correlations between musical aptitude or music training and linguistic skills in adult musicians and musically trained children. Musicians outperform non-musicians in syllable discrimination^1^ and in detecting speech in noise^2,3^. Furthermore, musical experience correlates with verbal memory in adults and children^4,5^ and with detection of prosody in children^6^. Children’s musical aptitude or the duration of their musical experience has been found to associate with reading skills^7,8^, vocabulary^9^ and phonemic awareness^8^, although there is also contradicting evidence^9^. The acquisition of foreign language sound structures both in adults^10,11^ and children^12^ has an association with musical – more specifically with rhythm perception – aptitude^13,14^, according to some studies. In addition, adult musicians have been found to detect pitch variations in an unknown foreign language better that non-musicians^15^.

While a lot of the studies are correlational, a growing body of evidence from behavioural research suggests that there are also causal links between music and language. The academic scores for second language rose faster for children who played an instrument during the six follow-up years, compared to non-musician children^16^. Two-year group music training – first twice and later once a week – improved children’s word segmentation skills more than painting training did^17^, and weekly instrumental lessons for 12 or 18 months also improved verbal memory in children and adolescents^5,18^. An intensive one-year music program affected positively reading and rapid naming skills of low-SES children^19^, while another study^20^ showed that daily music lessons lasting for a year enhanced kindergarten children’s phonological awareness. Additionally, a nine-month music training taking place twice a week affected positively children’s reading accuracy compared to a group receiving visual arts training^21^. Furthermore, twice-a-week music intervention lasting for seven months improved dyslexic children’s phoneme processing and reading skills more than painting intervention did^22^, and in line with this, Moreno *et al*.^23^ found that unlike painting training, twice-a-week music training for six months improved typically developing children’s reading abilities. Children’s phonological awareness was improved more by 20-week music and phonological programs than by sports program^24^, but at least one study^25^ failed to find a link between music and phonological awareness after thrice-a-week intervention lasting for five months. Overy^26^ reported that dyslexic schoolchildren benefitted from 15-week music intervention including three sessions a week as shown in phonological and spelling ability. Furthermore, a rhythm-focused music intervention including 19 sessions and lasting for two months enhanced 6–7-year-old poor readers’ literacy skills as much as the grapheme-phoneme intervention^27^, although it seems that adding musical elements in phonological training does not further enhance children’s early foreign language reading^28^. Finally, Moreno *et al*.^29^ showed that after computerized two-hour daily training for only 20 days, children in music listening group outperformed their peers in visual art group in verbal intelligence test.

To summarize: many types of music lessons have been found to affect children’s linguistic skills. The more intensive the intervention is, the faster the linguistic transfer effects are perceived.

The relationship between music training and intelligence has been a much debated issue during recent years. Music training^30–33^, and its duration^34^ have been positively associated with intelligence in children^31^, adolescents^30^ and adults^32,35^ in numerous studies. Forgeard *et al*.^9^ found a significant difference between children with and without musical training in non-verbal reasoning skills, as did Bergman-Nutley *et al*.^36^ in children and adolescents. Furthermore, Swaminathan *et al*.^37^ reported a correlation between non-verbal intelligence and musical aptitude. Nevertheless, the children who partake in music lessons come typically from more privileged backgrounds^38^ and – especially in correlational studies – this might distort the interpretation of the results.

However, some evidence for causal links between training of musical instrument and intelligence has also appeared. In the pioneering study by Schellenberg^39^, 6-year-old children were randomly assigned in groups taking lessons either in piano, singing or drama or not receiving any extra lessons. After one year of interventions, music groups outperformed the drama and control groups in overall intelligence and in most of the subtests. Other studies have also found evidence for causal links between music training and intelligence^40,41^, but the lack of active control groups limits the conclusions that can be reliably drawn from these reports. Even listening to enjoyable music has been found to enhance 10–11-year-old children’s spatial abilities^42^. However, as there is also contradictory evidence about the links between music and intelligence^29,43^ the issue still needs clarification.

Within the executive function domain, inhibitory control refers to the ability to deliberately resist automatic, or dominant processing when necessary^44^. While there are studies linking music training to executive functions in general^34,45,46^, only few of them focus on inhibitory control. In a recent study^47^, after attending to twice-a-week music training for only six weeks, small children scored better in inhibition test than their peers attending to spatial training with bricks that focused on problem-solving with spatial relationships. Additionally, children receiving intensive music training performed better by the end of the follow-up in a go/no-go task than the children in painting training group, along with an enhanced P2 event-related-potential component – suggested to reflect inhibitory control^29^. In contrast to these studies, Schellenberg^33^ did not find any advantage in inhibition subtest for musically trained group of children.

Overall, the evidence for causality between music training and inhibition is still scarce^45,48^, and more research is needed.

Many of the studies cited above have used time-consuming interventions that can rarely be implemented in kindergartens’ and schools’ weekly curricula and thus, are not available to all children, irrespectively of their background. Our interest here is to find implementations that could be beneficial for children with a low SES, connected in many studies to language and learning skills. Furthermore, the short interventions with pre- and post-tests may tell us whether music training has transfer effects on cognitive skills, but we need long follow-up studies to find out if such training truly affects developmental trajectories also in children from low-SES backgrounds. We wanted to study the regular Finnish music playschool, comprising 45-minute weekly group music lessons for toddlers and pre-schoolers, where a considerable part of parents take their children in Finland. Music playschool is taught by professional music educator who typically studies music and music pedagogy in a Bachelor’s or Master’s programme for 4–5 years and specializes in teaching small children. Importantly, in some cities music institutes provide these lessons also in kindergartens, which minimizes the efforts of parents, and thus, makes participating in music playschool more likely for families with a wider socio-economical background. Our data collection was conducted in altogether 26 kindergartens in Helsinki metropolitan area during two years, with a total of 66 children who participated either music playschool, dance playschool (active control group), or regular day care activities (passive control group).

Our hypothesis was that *music playschool enhances children’s linguistic skills*. We included in the test battery also tests for intelligence and inhibition to test if children participating in music playschool – in case they proved to have different developmental trajectory in linguistic skills – would differ from the other children also in these abilities, either in baseline or later during the follow-up. In addition to children not attending to any extra activity, we recruited as active controls children participating in dance lessons, organized in a similar manner as the music playschool.

## Results

Our results, based on several behavioural outcome measures, systematically show that music playschool enhances children’s linguistic skills. All the test scores are depicted in Table 1. Only the significant results are reported here; all the results are shown in Supplementary information, Tables S1–S4.

**Table 1:** Mean (standard deviation) values for children’s age at the beginning of the follow-up, mother’s education and all test scores. Scale for mother’s education 1–7: 1 = comprehensive school, 5 = lower university/bachelor’s degree, 7 = doctoral degree.

All (N = 66, female N = 41)			
	Mean (SD)	Minimum	Maximum
Age (months)	61 (3.6)	53	69
Mother’s education	4.8 (1.4)	1	7
***Phoneme processing***			
Test 1	26.3 (2.7)	20	33
Test 2	28.1 (3.6)	22	41
Test 3	29.2 (4.4)	21	44
Test 4	33.0 (5.3)	23	47
***Vocabulary***			
Test 1	12.1 (4.3)	4	26
Test 2	13.4 (4.9)	5	25
Test 3	15.2 (5.5)	6	29
Test 4	17.8 (4.9)	8	28
***Perceptual reasoning index***			
Test 1	27.4 (8.4)	15.0	49.5
Test 2	30.0 (8.2)	12.0	48.0
Test 3	32.4 (7.6)	16.5	49.5
Test 4	34.6 (7.2)	18.0	49.5
***Inhibition***			
Test 1	8.9 (2.7)	4	15
Test 2	9.1 (2.9)	5	15
Test 3	9.8 (3.0)	4	16
Test 4	10.0 (2.8)	5	15

### Phoneme processing

**Figure 1:**
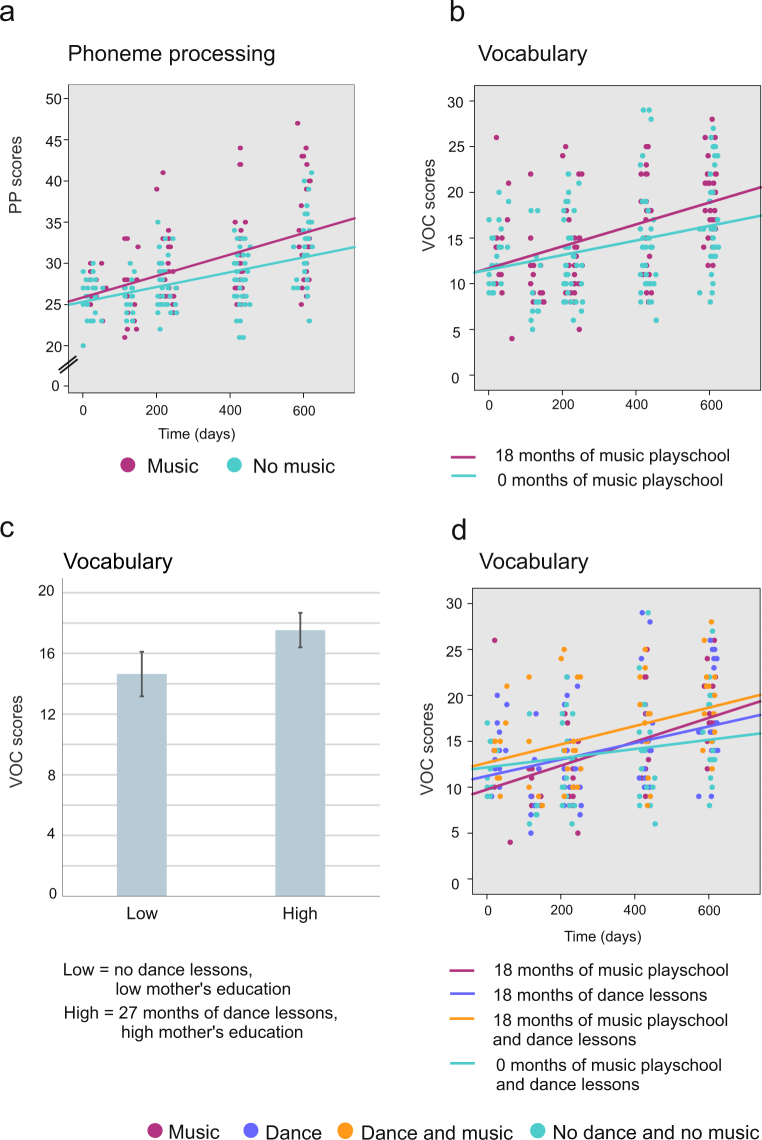
Individual test scores for *Phoneme processing* and *Vocabulary* for all four test times. The lines in (**a** and **b**) represent for illustration purposes the development of scores over time for children participating in music playschool either for 18 or 0 months and in (**d**) for children participating for 18 months either in music playschool or dance lessons, children participating in both activities for 18 months and children not participating in either activity. (**c**) The bars represent for illustration purposes the mean *Vocabulary* scores for children either not participating in dance lessons and having mother with low education (Low: scores 14.639, SEM = ±1.474) or participating in dance lessons for 27 months and having mother with high education (High: scores 17.534, SEM = ±1.138). Low mother’s education stands for 2 and high for 6 on the scale of 1 to 7.

*A linear mixed model growth curve* analysis for *Phoneme processing* revealed that the main effects of music playschool [F(1, 55) = 8.121, *p* = 0.006, parameter estimate = 0.095, indicating a rise of 0.095 points in scores per month of music playschool] and time [F(1, 177) = 142.00, *p* < 0.001, parameter estimate = 0.011, indicating a rise of 0.011 points in scores per day and 4.015 points in a year] were significant, as was the interaction between music playschool and time [F(1, 175) = 8.55, *p* = 0.004, parameter estimate = 0.0002, i.e., a month in music playschool increased the daily growing scores with additional 0.0002 points]. This indicates that for those children who participated in music playschool, improvement in *Phoneme processing* was significantly higher (for illustration purposes, see Fig. 1a). All the results are depicted in Supplementary Information, Table S1.

### Vocabulary

For *Vocabulary*, the main effect of time [F(1, 176) = 95.535, *p* < 0.001; parameter estimate = 0.010, indicating a rise of 0.010 points in scores per day and 3.65 points in a year] was significant, as was the interaction between music playschool and time [F(1, 174) = 7.30, *p* = 0.008, parameter estimate = 0.0002, i.e. a month in music playschool increased the daily growing scores with additional 0.0002 points]. This indicates that for those children who participated in music playschool improvement in *Vocabulary* was significantly higher (for illustration purposes, see Fig. 1b). Furthermore, there was a significant dual contribution of dance lessons and mother’s education [F(1, 55) = 4.95, *p* = 0.030, parameter estimate = 0.061, indicating that dual contribution of each month in dance lessons and one step higher mother’s education increased the test scores with 0.061 points], children with more dance lessons and higher mother’s education scoring higher in *Vocabulary* (for illustration purposes, see Fig. 1c). Additionally, a significant three-way-interaction for dance lessons, music playschool and time [F(1, 175) = 10.77, *p* = 0.001] was found (for illustration purposes, see Fig. 1d). However, the estimate for interaction between music playschool, dance lessons and mother’s education was negative (parameter estimate = −2.036 * 10^−5^) and very small. All the results are depicted in Supplementary Information, Table S2.

### Perceptual reasoning index and Inhibition

**Figure 2:**
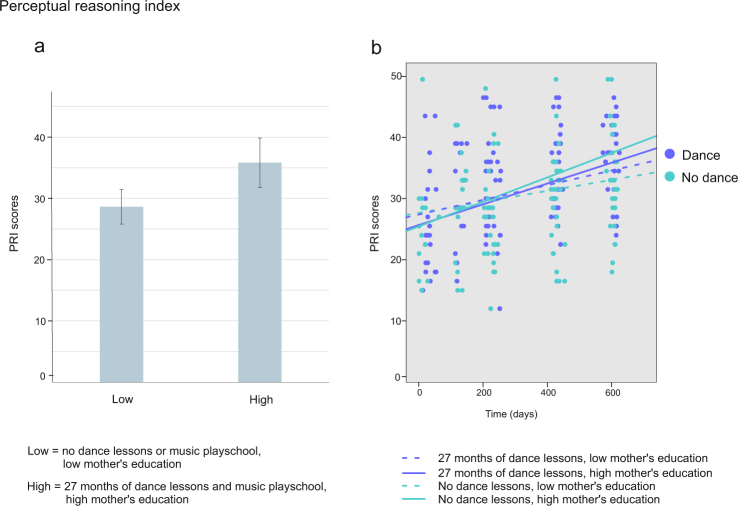
Mean and individual scores for *Perceptual reasoning index*. (**a**) The bars represent for illustration purposes the mean *Perceptual reasoning index* scores for children either not participating in dance lessons and having mother with low education (Low: scores 28.626, SEM = ±2.823) or participating in dance lessons for 27 months and having mother with high education (High: scores 35.808, SEM = ±4.024). (**b**) Individual test scores for all four test times. The lines represent for illustration purposes the development of the scores for children participating in dance lessons for 27 or 0 months and having mother’s with high or low education. Low mother’s education stands for 2 and high for 6 on the scale of 1 to 7.

For *Perceptual reasoning index* (i.e. the sum Block design and Matrix reasoning scores), the main effect of time [F(1, 176) = 99.42, *p* < 0.001, parameter estimate = 0.014, indicating a rise of 0.014 points in scores per day and 5.11 points in a year] was significant, as was the interaction between music playschool, dance lessons and mother’s education [F(1, 56) = 6.72, *p* = 0.012, parameter estimate = 0.009, indicating that a month in music playschool and dance lessons and one step higher mother’s education increased the test scores with 0.009 points] indicating that for those children who participated more in music playschool and dance lessons and had higher maternal education the scores in *PRI* test were significantly higher (for illustration purposes, see Fig. 2a). Furthermore, interaction between dance lessons, time and mother’s education [F(1, 175) = 4.32, *p* = 0.039, parameter estimate 0.0001] was significant, but theoretically difficult to interpret (for illustration purposes, see Fig. 2b). All the results are depicted in Supplementary Information, Table S3.

In addition to main effect of time [F(1, 179) = 9.10, *p* = 0.003, parameter estimate = 0.002, indicating a rise of 0.002 points in scores per day and 0.73 points in a year], there were no significant main effects or interactions for the *Inhibition* subtest. All the results are depicted in Supplementary Information, Table S4.

## Discussion

The current data indicate that attending to weekly music playschool enhanced the development of 5–6-year-old children’s phoneme processing skills and vocabulary. Dance lessons did not show similar effects on these skills. The difference between children’s development became apparent during our two-year follow-up. Thus, our study is in line with previous research^17–24,29^ suggesting that the more intensive the intervention, the sooner the possible transfer effects appear.

Previously, phonological awareness – closely linked with phoneme processing – has been found to benefit from group music interventions lasting from one year^20^ to only 20^24^ or 15 weeks^26^. Nevertheless, the kindergarteners attending to one-year intervention^20^ received a substantial amount of music lessons during the year – 45 minutes each day, and 20-week intervention^24^ included daily 10-minute sessions in small groups led by trained research assistants. Counting the weekly minutes, the 20-week intervention did not include much more music than in our study, so the decisive factor here might be the regularity and frequent repetition of musical activities. Intensity is probably also the reason for Moreno *et al*.^29^ finding effects of computerized group music training on pre-school children’s vocabulary scores only after twenty days of training. The difference between the effective duration of former and the present interventions may also lie in the differences between kindergarten curricula in different countries. In Finland where most day-care is provided and supported by municipalities, musical activities are an inherent part of all kindergartens weekly program. Thus, the benefits of an *additional* music playschool provided by professional music playschool teachers in some kindergartens – like in our study –might be revealed only after longer interventions. The realization of *self-provided* music sessions, naturally, varies between kindergartens according to the skills and interests of the personnel.

To our knowledge, the present study is the first that uses dance – activity closely related to music training – as control intervention. Notably, we found no consistent evidence for a benefit of dance lesson on any of our linguistic outcome measures even though, like music playschool, dance lessons also include musical elements such as synchronizing one’s actions with rhythm, listening to music and expressing its emotional content. Both are also social acts where children can enjoy doing things together. So, what is different? In music playschool children produce different pitches mostly by singing but also with simple instruments, and thus practice to differentiate frequency content of sounds. This active sound production rehearsal – rather than just more passive listening of music or moving to music – might contribute to the development of phoneme processing, which requires accurate processing of frequency information. Singing might also be the factor that affects the vocabulary learning: songs introduce new words and consolidate the meaning of more familiar ones in the developing brain. However, in a study by Moreno *et al*.^29^ the training was mostly auditory and not productive and, apparently, did not include much singing. So, vocabulary knowledge and listening skills may somehow be linked via shared cognitive processing, not yet fully understood. Naturally, the effects of music activities on language development could also be mediated via e.g., motivation. Children attending to music playschool might become more motivated in for instance, rhyming and singing by themselves and this could lead to enhancement of linguistic skills.

Interestingly, children who had experience in both music playschool and dance lessons did not seem to get an advantage from these activities in their vocabulary development. This might be due to them scoring relatively high already in the beginning of our follow-up, and possibly facing a ceiling effect along the follow-up. In our study, children who attended music playschool and had lower scores in the first measurements reached this better scoring group by the end of the follow-up. Thus, our result supports the suggestion that music activities might benefit more children who are not performing especially well in linguistic tasks, at least within the inspected age range of 5 to 6 years.

Contrary to recent findings^49^, no different developmental trajectories were revealed between the children in the *Inhibition* test. Thus, it seems that the linguistic skills tested here are not mediated via strong inhibitory control. Furthermore, this result emphasizes that children belonging to different groups did not differ in their inhibition skills from each other. Our results of non-verbal intelligence, by contrast, are not straightforward. Children from more educated families tend to have more extra-curricular activities and to do better in intelligence tests than their less privileged peers, as has been found in several studies^50^. Although our results did reveal a statistically significant three-way interaction between dance lessons, mother’s education and time on PRI scores, this result does not allow any straightforward interpretation about the impact of these factors on children’s development. Furthermore, it was the weakest observed result in the whole study. Why this result is apparent with dance lessons but not with music playschool might be due to the fact that in our sample the children who participated in dance lessons had mothers with slightly higher education than the other children.

In connection with our other results, this further strengthens the suggestion that children participating in music playschool did not differ from others in their intellectual capacities, and their better performance in linguistic skills is not strongly linked with more general cognitive skills, e.g. intelligence or executive functions. In addition, the present results do not support previous studies^39,40^ suggesting that music training causes enhancement in intelligence scores.

In order to promote policies contributing to social equality, it is crucial to study the possible effects of easily applicable music interventions compared to training programs that are difficult to implement in children’s curricula. All parents do not have financial or social resources for taking their children to music lessons, possibly several times a week, and this further increases the gap between children coming from different socio-economic backgrounds. It is particularly promising to find that inexpensive traditional music playschool widely used in Finland has transfer effects on children’s linguistic abilities, skills that are massively contributing to their future academic achievements and overall well-being. The additional benefit of weekly music playschool – in comparison with e.g., phonemic training^24,27,28^ – comes from the enjoyment most children get from singing and playing together. Moreover, in addition to being fun, joint music making seems to promote social skills^51–53^, which further adds up to reasons for encouraging kindergartens and schools – and the society behind them – to provide such group music activities for children.

Yet, studies comparing children with or without music training are often criticized for not using randomized controlled trial designs. Nevertheless, as Habibi *et al*.^54^ pointed out, it is difficult to get unmotivated children and their parents to continue participation in a longitudinal study for a long period of time, which is known to be needed for the learning to occur^55^. In addition, even randomized controlled trials cannot overcome the fact that children with higher socio-economical backgrounds are more likely to have support for their musical training than other children, if they happen to be randomized into the music training group. Therefore we argue that the use of community based samples in such extensive follow-up studies is a well-justified choice, as long as some essential background information of the children is collected in the study, which later on can also be taken into account in statistical analyses^56^. In our view, in fact, community-based studies offer an optimal compromise between full randomization designs, adopted from animal studies, and studies comparing children who are involved in music or any other hobbies based on their initiative. Here, the involvement is voluntary but does not require additional resources in terms of time, travel, or funding.

Of relevance here is that according to recent views, socio-economical and other environmental factors are appended by a genetic contribution when motivation to continue music and other hobbies is concerned. These genetic factors, in turn, will thus *indirectly* affect the degree of expertise acquired and the amount of possible transfer to other domains^57^. Unfortunately, based on studies with humans, it would not be feasible to determine the relative importance of environmental vs. genetic factors in a given skill. However, with animal models this is possible and in fact, with regard to bird song learning, this has been very recently conducted^58^. Opposite to the birds (Bengalese finches) in a computerized setting, the birds in an enriched live setting showed an increase in the influence of experience on learning song tempo and, in parallel, a significant decrease in the influence of genetics. In the current context of music and its transfer effects this study on birds is of upmost importance by showing the possibilities of natural interaction and face-to-face contact to optimize the learning outcome and reducing negative genetic influences. In other words, it is possible that the differences in genetic predisposition to enjoy music and benefit from it could be overdriven by high quality of teaching in naturalistic socially engaging environments.

## Methods

### Participants

Original sample constituted of 84 children all going to municipal kindergartens in Helsinki metropolitan area. After excluding all bilingual children (N = 15), one child with special educational needs, and two drop-outs after first test, we ended up with total N = 66 (female N = 41). The caregivers filled a questionnaire about children’s family background and extra-curricular activities in the beginning of the study, and further informed about the possibly changed status of these activities in the end of the follow-up. Children’s mean age at the beginning of the follow-up was 61 months (SD 3,6), and they were followed for two school years. As part of the children changed the kindergarten after one year, altogether 26 different kindergartens were recruited to the study. Of these, nine offered the children a possibility to attend to music playschool provided by an outside music institute and eight offered dance lessons provided by a dance institute. None of the kindergartens offered both music playschool and dance lessons. Both of these activities took place during the day-care at kindergarten premises. The passive control children went to nine kindergartens that did not offer any activity provided by an outside institute. The music and dance institutes had offered their lessons to several kindergartens in this particular city in Helsinki metropolitan area and the kindergartens had decided for themselves if they wanted to provide such activity to the children. The kindergartens with dance lessons were located in areas with slightly higher socio-economic status than the kindergartens with music playschool or with neither activity. Otherwise, there were no differences in e.g., the level of teachers’ education or geographical or socio-economical areas between the kindergartens.

As we studied a community based sample and interventions provided by music and dance institutes, we were not able to restrict children’s participation in music playschool or dance lessons in any way. Some children had started music playschool or dance lessons already before the start of the research and some partook the other extra-curricular activity provided by music or dance institute outside the kindergarten. To take this into account in the analyses, we added these activities, reported by parents, in the total amount of months of participation in music or dance training for each child. Thus, we did not compare groups, but predicted the test scores with the amount of months the children had spent in music playschool or/and dance lessons by the end of our follow-up. By the end of the study of all children participating, 28 (female N = 19) had been in music playschool for at least some time after turning three (variation 9–36 months) and 32 (female N = 26) in dance lessons, (variation 1–40 months). Twenty kindergarteners (female N = 8) had not participated in either.

The guardians signed a written informed consent and the children gave their verbal assent before the experiment. The experiment protocol was approved by The Ethical Committee of the Humanities and Social and Behavioural sciences in the University of Helsinki, Finland, and the experiments were carried out in accordance with the committee’s guidelines and regulations as well as with those of Helsinki declaration.

### Music playschool and dance lessons

According to Finnish music playschool tradition, whether it is organized in kindergartens or in music institutes’ own premises, lessons consist of singing, playing games, rhyming, playing simple instruments (xylophones, small drums etc.), body percussions aimed at improving fine and gross motor skills, along with listening and moving to music. Each lesson is slightly different but they all include musical elements like singing and synchronizing one’s motor actions with the beat and with other children. The dance lessons in our study – and for small children in general – constituted mainly of practicing basic motor skills, rhythms, some elementary improvisation and group movement training. Exercises aim to develop children’s percept of their own body, rhythm and space, along with teaching them to act in a group. Like with music playschool, the individual lessons taught by a professional dance teacher specialized in teaching small children differed somewhat in details but included the same elements throughout the year. Typically, practice at home is neither encouraged nor prohibited, and it was not controlled in our study. Both music playschool and dance lessons were held in groups of 8–12 children, lasted 45 minutes and took place once a week, 30 times a year. The music playschool and the dance lessons cost 100€/semester but are free for the families with very low income.

As musical activities are part of Finnish kindergartens’ curricula, the personnel working with the studied children answered each year of the follow-up a questionnaire about the musical activities in kindergarten, not including the professionally guided music playschool and dance lessons in focus of our study. The monthly amount (minutes) of kindergarten group’s own music activities (average over two years) varied substantially between children, depending on the group the child was in [mean = 163; SD = 82; max = 390; min = 60]. The kindergartens that provided music playschool or dance lessons did not differ from each other or from the kindergartens not having either of the extra-curricular activities in respect of their averaged amounts of self-provided music activities [χ^2^(6) = 6.841, *p* = 0.336].

### Neurocognitive assessments

During the two years, children were tested four times with *Phoneme processing* and *Inhibition* subtests from NEPSY II developmental neuropsychological test battery^59^ along with *Vocabulary*, *Block design* and *Matrix reasoning* subtests from WISC IV^60^, a intelligence scale for children. *Block design* and *Matrix reasoning* were combined according to instructions in WISC-IV to form *Perceptual Reasoning Index (PRI*) (Table 1). The internal reliability and validity of NEPSY II are very good^61,62^ and it is widely used in clinical work in Finland. WISC IV has a very high internal reliability and even though it’s validity has raised some conversation^63^, it correlates highly with other intelligence scales for children and is the most widely used intelligence batteries in the world^60,63^.

All the tests were rehearsed before the experiment with children, according to the test guidelines.

*Phoneme processing* (*PP*) subtest measures phonological awareness and auditory memory. In the first section of the test the child saw pictures of objects and heard names for them. The experimenter pronounced then a phoneme combination that was included in one of the object names and the child picked the object the phoneme combination belongs to. In the next section the child was asked to remove a phoneme or combination of phonemes from the uttered word and say the resulting word (“Say/tak:a/, ‘fireplace’. Then say the same word without/t/”, the right answer being/ak:a/, ‘an old woman’). In the final section the child is asked to replace a phoneme with a given one (“Say/helmi/, ‘pearl’. Now say the same word but replace/i/with/a/”. The right answer is/helma/, ‘hem of skirt’). Testing was stopped after six consecutive wrong answers.

*Vocabulary (VOC)* test measures child’s verbal knowledge and ability to format concepts. The experimenter presented words orally and the child defined the words that become increasingly difficult. The sophistication of definition was scored with 0, 1 or 2 points. E.g., “cow” could be answered “animal”, “mammal”, “domestic animal” (2 points), “you can milk cows”, “says moo” (1 point), or “eats grass”, “calf” (0 points). Testing was stopped after five consecutive answers given zero points.

*Block design* and *Matrix reasoning* are subtests of The Perceptual Reasoning Index’s section of WISC-IV. Block design measured visuospatial skills. The child formed patterns with red-and-white blocks according to a displayed model, and this performance was timed. In Matrix reasoning test children were shown an array of pictures with one missing square, and were asked to select from five options the picture that fits the array. The test measured fluid reasoning skills. In the present study, we combined these tests according to instructions in WISC-IV to form Perceptual Reasoning Index (*PRI*) that acts as an indication of non-verbal intelligence.

*Inhibition* (*INH*) test measures two kinds of skills linked to executive functions, namely inhibition and task shifting. The child was asked to tell the orientations of arrows or their opposites according to the rules given before each take. The test was then repeated with circles and squares.

All the neurocognitive tests were conducted in the kindergarten premises during day-care in a separate room with only the participant and the experimenter present. The testing took 45–60 minutes. Children got cookies and soft drink during breaks (with the permission from the parents). In analyses we used raw scores for *PP* and *VOC*, and standard scores for *PRI* and *INH*.

### Statistical analyses

There was considerable variation in the first test times. To take this into account we conducted the analyses with linear mixed models, specifically, with *linear* *growth curve model*^64^. In addition, the mixed model analysis allows the use of all available data despite the missing values. Model was created separately for each neurocognitive test. Centred values for time, duration of music playschool and dance lessons (months by the end of the follow-up) and mother’s education, along with all the interactions between these acted as predictors, and the test scores as dependent variables. Time was indexed by children’s individual test days. Thus, the first test day was marked as 1, and e.g., a test day a week later was marked as 8. As a result we got a time line were the first tests were conducted between days 1–150, second between days 201–255, third 412–455 and the fourth between days 573–624.

Random intercept model was used in all analyses. On the basis of Schwarz’s Bayesian Criterion (BIC), compound symmetry was chosen as the covariance structure. All the analyses were conducted with SPSS 24 (IBM Corporation, NY, USA). Only the significant main effects and interactions are reported in the results, and tables for all the results are in Supplementary Information (SI). We checked by visual inspection that the residuals were approximately normally distributed and did not violate the assumption of homoscedasticity. Alpha level was set at p < 0.05.

### Data availability

The datasets generated and analysed during the current study are available from the corresponding author on reasonable request.

## Electronic supplementary material

Supplementary Information: Main effects and interactions
