# Supplementary material for: Music playschool enhances children’s linguistic skills
Source: Sci Rep. 2018 Jun 8;8:8767. doi: 10.1038/s41598-018-27126-5 (PMC5993803; doi:10.1038/s41598-018-27126-5)
Supplement: Supplementary file 1 — Supplementary Information: Main effects and interactions [file 41598_2018_27126_MOESM1_ESM.pdf]

Supplementary information

Table S1 The main effects and interactions for *Phoneme processing* test scores. The significant results are marked in bold.

Main effects and interactions	*F*	*p*
Time	*F(1, 177)*=142.997	***p*< .001**
Music playschool	*F(1, 55)*=8.121	***p*=.006**
Dance lessons	*F(1,55)*=3.232	*p*=.078
Mother's education	*F(1,55)*=.584	*p*=.448
Music playschool x Time	*F(1, 175)*=8.547	***p*=.004**
Dance lessons x Time	*F(1,176)*=1.652	*p*=.200
Mother's education x Time	*F(1,176)*=.779	*p*=.379
Music playschool x Mother's education	*F(1,55)*=.898	*p*=.347
Dance lessons x Mother's education	*F(1,55)*=.355	*p*=.554
Music playschool x Dance lessons	*F(1,55)*=2.884	*p*=.095
Music playschool x Dance lessons x Time	*F(1,176)*=.678	*p*=.411
Music playschool x Time x Mother's education	*F(1,176)*=1.815	*p*=.180
Dance lessons x Time x Mother's education	*F(1,176)*=2.284	*p*=.133
Music playschool x Dance lessons x Mother's education	*F(1,55)*=1.836	*p*=.181

Table S2 The main effects and interactions for *Vocabulary* test scores. The significant results are marked in bold.

Main effects and interactions	*F*	*p*
Time	*F(1, 176)*=95.535	***p*< .001**
Music playschool	*F(1,55)*=2.722	*p*=.105
Dance lessons	*F(1,55)*=2.667	*p*=.108
Mother's education	*F(1,55)*=1.795	*p*=.186

Music playschool x Time	*F(1, 174)*=7.301	***p*=.008**
Dance lessons x Time	*F(1,175)*=.019	*p*=.892
Mother's education x Time	*F(1,175)*=.004	*p*=.950
Music playschool x Mother's education	*F(1,55)*=.097	*p*=.757
Dance lessons x Mother's education	*F(1, 55)*= 4.953	***p*=.030**
Music playschool x Dance lessons	*F(1,55)*=2.540	*p*=.117
Music playschool x Dance lessons x Time	*F(1, 175)*= 10.767	***p*=.001**
Music playschool x Time x Mother's education	*F(1,175)*=2.101	*p*=.149
Dance lessons x Time x Mother's education	*F(1,175)*=3.662	*p*=.057
Music playschool x Dance lessons x Mother's education	*F(1,55)*=2.511	*p*=.119

Table S3 The main effects and interactions for *Perceptual reasoning index* scores (i.e. the sum Block desing and Matrix reasoning scores). The significant results are marked in bold.

Main effects and interactions	*F*	*p*
Time	*F(1, 176)*=99.424	***p*< .001**
Music playschool	*F(1,56)*=.112	*p*=.739
Dance lessons	*F(1,56)*=.581	*p*=.449
Mother's education	*F(1,56)*=.000	*p*=.991
Music playschool x Time	*F(1,175)*=.545	*p*=.461
Dance lessons x Time	*F(1,175)*=.725	*p*=.396
Mother's education x Time	*F(1,175)*=1.062	*p*=.304
Music playschool x Mother's education	*F(1,56)*=.018	*p*=.893
Dance lessons x Mother's education	*F(1,56)*=.436	*p*=.512
Music playschool x Dance lessons	*F(1,56)*=.056	*p*=.813

Music playschool x Dance lessons x Time	*F(1,175)*=1.817	*p*=.179
Music playschool x Time x Mother's education	*F(1,175)*=.497	*p*=.482
Dance lessons x Time x Mother's education	*F(1, 175)*=4.320	***p*=.039**
Music playschool x Dance lessons x Mother's education	*F(1, 56)*=6.721	***p*=.012**

Table S4 The main effects and interactions for *Inhibition* test scores. The significant results are marked in bold.

Main effects and interactions	*F*	*p*
Time	*F(1, 179)*=9.103	***p*=.003**
Music playschool	*F(1,55)*=.010	*p*=.922
Dance lessons	*F(1,55)*=.199	*p*=.657
Mother's education	*F(1,55)*=1.105	*p*=.298
Music playschool x Time	*F(1,176)*=1.536	*p*=.217
Dance lessons x Time	*F(1,178)*=.963	*p*=.328
Mother's education x Time	*F(1,177)*=.124	*p*=.725
Music playschool x Mother's education	*F(1,55)*=3.308	*p*=.074
Dance lessons x Mother's education	*F(1,54)*=.618	*p*=.435
Music playschool x Dance lessons	*F(1,54)*=.163	*p*=.688
Music playschool x Dance lessons x Time	*F(1,177)*=.016	*p*=.900
Music playschool x Time x Mother's education	*F(1,177)*=.009	*p*=.925
Dance lessons x Time x Mother's education	*F(1,177)*=1.246	*p*=.266
Music playschool x Dance lessons x Mother's education	*F(1,54)*=.001	*p*=.973
